# Overexpression of *NtDOG1L-T* Improves Heat Stress Tolerance by Modulation of Antioxidant Capability and Defense-, Heat-, and ABA-Related Gene Expression in Tobacco

**DOI:** 10.3389/fpls.2020.568489

**Published:** 2020-10-30

**Authors:** Xiaoyan Dai, Yingfeng Wang, Yanchun Chen, Hongchen Li, Shixiao Xu, Tiezhao Yang, Xiaoquan Zhang, Xinhong Su, Zongliang Xia

**Affiliations:** ^1^College of Tobacco Science, Henan Agricultural University, Zhengzhou, China; ^2^Sanmenxia Tobacco Company, Sanmenxia, China; ^3^College of Life Science, Henan Agricultural University, Zhengzhou, China

**Keywords:** heat stress, promoter activity, antioxidant enzyme, gene expression, transgenic tobacco, DOG1

## Abstract

Drought and heat stresses are two major environmental stress factors that severely threaten crop growth and productivity. Plant *delay of germination 1-like* (*DOG1L*) family genes play important roles in various developmental processes and stress responses. In our previous study, a tobacco *DOG1L* gene (*NtDOG1L-T*) was found to regulate seedling growth and drought response. Unfortunately, the role of *DOG1L* genes in heat stress response is yet to be studied. Here, we present data supporting the role of *DOG1L* genes in heat stress and possible underlying molecular mechanisms. Transcript levels of *NtDOG1L-T* were rapidly induced by heat or abscisic acid (ABA) treatment. Furthermore, *NtDOG1L-T* promoter activity was markedly activated by ABA or heat stress, as revealed by histochemical staining in transgenic tobacco seedlings. Overexpression of *NtDOG1L-T* in transgenic lines improved heat stress tolerance. The *NtDOG1L-T*-transgenic plants exhibited lower levels of reactive oxygen species (ROS) and lipid peroxidation but higher antioxidant enzyme activities in response to heat stress. Furthermore, transcript abundance of some defense-, heat-, and ABA-responsive marker genes was significantly upregulated, as shown by reverse transcription quantitative PCR (qPCR) in these transgenic plants. In conclusion, *NtDOG1L-T* positively regulates heat stress tolerance possibly by modulation of antioxidant capability and defense-, heat-, and ABA-related gene expression in tobacco. This study may provide valuable resource for the potential exploitation of *DOG1Ls* in genetic improvement of heat stress tolerance in crops.

## Introduction

With aggravation of global warming, heat stress caused by high temperature has become an increasingly serious problem in agricultural production worldwide (Tubiello et al., 2007). Heat stress leads to excessive reactive oxygen species (ROS) accumulations, membrane lipid peroxidation, and metabolic disturbance, even cell death in the plant (Iba et al., 2002; Choudhury et al., 2017). Thus, heat stress adversely affects development, yield, and quality of crops. Over the long time of evolution, plants have developed multifaceted adaptive mechanisms, in which numerous stress defense genes (or proteins) are activated to cope with the heat stress (Ohama et al., 2017). Such functional genes mainly encode signaling molecules such as mitogen activated protein kinase (MAPK) cascades, cellular protective enzymes such as peroxidase (POD), transcription factors such as heat shock transcription factors (HSFs), and cellular stress proteins such as heat shock proteins (HSPs). Some of these genes have been reported to protect plant cells from damage through sustaining cellular ROS homeostasis or regulating downstream signaling pathway under heat stress (Jacob et al., 2017; Raja et al., 2017; Zhang et al., 2018). It was reported that the abscisic acid (ABA)-deficient and ABA-insensitive *Arabidopsis* mutants were susceptible to heat stress, whereas overexpression of the ABA-responsive element-binding protein (AREB) enhanced thermotolerance (Suzuki et al., 2016), demonstrating that ABA signaling plays a positive role in heat stress response. Nowadays, majority of the genes identified have great potential to improve heat stress tolerance in crop plants (Ul Haq et al., 2019). Thus, it is pivotal for crop genetic improvement to identify more stress-tolerant genes and explore their exact roles.

Delay of germination 1 (DOG1) is a plant-specific protein, which contains an AHG-binding motif and a heme-binding domain, but has unknown biochemical function. The first *DOG1* gene was identified by genetic analysis of a major quantitative trait locus for increased seed dormancy in *Arabidopsis* (Bentsink et al., 2006). Since then, a great number of studies have been focused on regulatory mechanisms of the *DOG1* in seed dormancy and germination in *Arabidopsis*. It is reported that *Arabidopsis DOG1* regulates seed germination in a temperature- and gibberellin-dependent manner (Graeber et al., 2014). Interestingly, several reports have shown that the *DOG1* controls seed dormancy *via* formation of DOG1-PP2C phosphatase complex in the ABA signaling pathway (Née et al., 2017; Nishimura et al., 2018). Most recently, the *Arabidopsis DOG1* has been found to be involved in controlling seed dormancy and germination through ethylene (ET) signaling (Li et al., 2019). Also, Bryant et al. (2019) have found that *Arabidopsis DOG1* is activated by the basic LEUCINE ZIPPER TRANSCRIPTION FACTOR67 (bZIP67) during seed dormancy establishment (Bryant et al., 2019). These results suggest that *DOG1* is an important regulator in seed dormancy and germination in *Arabidopsis*.

In recent years, increasing evidence has shown that *DOG1* is required for multiple developmental processes and stress responses beyond seed dormancy in *Arabidopsis* (Teng et al., 2008; Dekkers et al., 2016; Huo et al., 2016; Yatusevich et al., 2017). For instances, Huo et al. (2016) showed that *Arabidopsis DOG1* could regulate seed dormancy and flowering time by affecting levels of miR156 and miR172 (Huo et al., 2016). That year, Dekkers et al. (2016) also reported a new role for *DOG1* in seed development using genetic analyses of *ABSCISIC ACID INSENSITIVE 5* (*ABI5*) and *ABI3* mutants in *Arabidopsis* (Dekkers et al., 2016). Apart from developmental processes, DOG1 also participates in stress responses in *Arabidopsis*. It was evidenced that decrease of antisense *DOG1* RNA (*asDOG1*) resulted in high levels of *DOG1* expression and enhanced drought tolerance in *Arabidopsis* (Yatusevich et al., 2017). In addition, *Arabidopsis* DOG1 has shown to be involved in ABA-mediated sugar signaling pathway by affecting *ABI4* expression (Teng et al., 2008; Vishwakarma et al., 2017). These findings indicate that *DOG1* plays vital roles in developmental and stress responses in *Arabidopsis*.

In crop plants, functional studies of *DOG1-like* (*DOG1L*) genes are still in infancy. It was reported that cereal *DOG1L* genes shared similar structural features among rice, wheat, barley, maize, and sorghum and had conserved roles in seed dormancy control in transgenic plants (Ashikawa et al., 2013, 2014). Common tobacco (*Nicotiana tabacum*, genome *TTSS*) is an allotetraploid species, which originates from interspecific hybridization of *Nicotiana sylvestris* (2*n* = 24, genome *SS*) and *Nicotiana tomentosiformis* (2*n* = 24, genome *TT*) about 200,000 years ago (Sierro et al., 2014). More importantly, tobacco is a global commodity with great economic value (Sierro et al., 2014). Nowadays, environmental stresses such as drought, heat, and chilling are still major limiting factors for productivity and quality of tobacco (Su et al., 2017). Recently, we have discovered a novel drought-responsive gene (named *NtDOG1L*), which encodes a DOG1-like protein by transcriptome analyses. Further evidence showed that the tobacco *DOG1L* was involved in drought tolerance *via* transgenic overexpression approach (Zhang et al., 2019). Unfortunately, the role of *DOG1L* genes in heat stress response is yet to be studied. Here, we further investigate its heat stress response and possible function mechanisms using the transgenic tobacco lines.

## Materials and Methods

### Plant Materials, Growth Conditions, and Stress Treatments

*Nicotiana tabacum* cv. Ws38 [wild type (WT)] and *NtDOG1L-T* transgenic lines (background Ws38) were used in this study. The *DOG1L-T* transgenic tobacco lines were produced as described previously (Zhang et al., 2019). Briefly, the CDS fragment of the *NtDOG1L-T* was subcloned into the binary vector pWM101 downstream of the CaMV 35S promoter. The resulting construct (pWM101-35S:NtDOG1L-T) was introduced into *Agrobacterium tumefaciens* strain *GV3101*, which was transformed into the tobacco cv. *WS38 via* the *Agrobacterium*-mediated leaf disc transformation method. Positive transgenic lines were screened and further identified by PCR and quantitative PCR (qPCR). Three independent lines (OE-5, OE-6, and OE-9) with high transcript levels were used for further analysis.

Tobacco seeds were germinated and grown on MS medium in a culture chamber at 25°C with a photoperiod of 16/8 h light/dark. After 10 days, these seedlings were transplanted into pots with a mixture of vermiculite and soddy soil (1:1) for cultivation in the growth room. The seedlings were irrigated weekly with Hoagland’s nutrient solution as described previously (Zhang et al., 2019). For heat and ABA treatments, 2-week-old tobacco seedlings were maintained at 45°C for heat stress (65% relative humidity) or were sprayed by 50 μM ABA at 25°C in growth chambers, and seedlings were sampled separately at various time points after each treatment. During heat stress, the pots with the nutrient soil were covered with nylon membranes to reduce water loss and avoid a dehydration stress of the seedlings. Harvested samples were immediately frozen in liquid nitrogen and then stored at −85°C for gene expression analyses.

### Tobacco *DOG1L-T promoter:GUS* Fusion Vector Construction and Transient GUS Expression Assays Under ABA or Heat Stress

The *NtDOG1L-T* promoter fragment (about 1.4 kb) was amplified by PCR with corresponding primers (Supplementary Table S1). Subsequently, the fragment was subcloned into the pCAMBIA1381 vector containing a *β-glucuronidase* (*GUS*) reporter with *Eco*RI/*Hin*dIII restriction sites and confirmed by sequencing.

Transformation and culture of the *Agrobacterium* harboring the recombinant plasmid (*pDOG1L-T-Pro:GUS*) were conducted as described by Xu et al. (2019). Then, the sterile cultured tobacco seedlings at 5- or 6-leaf stage were infiltrated by *A. tumefaciens* with the *promoter:GUS* construct. After 12 h the infiltrated seedlings were exposed to heat (45°C) or ABA (50 μM) treatment for 2 h, and then 6–8 seedlings for each treatment were collected for GUS histochemical staining. The infiltrated seedlings in MS plates under normal conditions were treated as controls. The transient GUS expression assays were carried out using a protocol described previously (Xu et al., 2019).

### Histochemical GUS Staining, Imaging, and Quantification

GUS staining assay was conducted as previously described (Xu et al., 2019). Briefly, tobacco seedlings were firstly incubated in GUS staining buffer (100 mM sodium phosphate, 10 mM EDTA, 1 mM 5-bromo-4-chloro-3-indolyl-β-D-glucuronic acid, and 0.5 mM potassium ferrocyanide) at 37°C for 12 h. Then, the seedlings were destained in 70% ethanol for 12 h, and imaged using a ZEISS upright light microscope equipped with a camera. Finally, quantification of GUS signal in aerial parts of the stained seedlings was done using ImageJ 1.41 software^1^ as described previously (Béziat et al., 2017).

### Heat Stress Tolerance Analysis of Tobacco *DOG1L-T* Transgenic Plants

The WT and transgenic tobacco lines (OE-5, OE-6, and OE-9) were cultured in MS plates under a 16/8 h light/dark cycle at 25°C in culture chamber for 2 weeks, and then the seedlings were transferred to pots filled with nutrient soil in a growth room where they were regularly cultivated for additional 6 weeks. And then, 8-week-old WT and transgenic lines were transferred to a culture chamber and subjected to a heat stress treatment (45°C) for 24 h. The corresponding WT and transgenic lines grown in the other chamber at 25°C were treated as controls. After that, phenotypes of treated and control plants were photographed, and electrolyte leakage and chlorophyll content in stressed or control plants were determined following the procedure described previously (Zhang et al., 2019). These experiments were performed in three biological replicates.

### Determination of Stress-Related Physiological and Biochemical Parameters

Eight-week-old transgenic lines and WT tobacco plants were transferred to a growth chamber and exposed to heat stress (45°C) for 24 h. Meanwhile, the corresponding WT and transgenic plants were grown in the other chamber under normal conditions as controls. After treatments, leaf samples were immediately taken for measurements of H_2_O_2_ and malondialdehyde (MDA) contents and major antioxidant enzyme activities, including superoxide dismutase (SOD), catalase (CAT), and POD in stressed and control plants, as described by Zhang et al. (2019). Accumulations of O_2_^−^ and H_2_O_2_ in leaf discs from treated or control plants were detected by nitro blue tetrazolium (NBT) and 3,3'-diaminobenzidine (DAB) staining, respectively as described by Sun et al. (2019).

### Reverse Transcription Quantitative PCR

The reverse transcription qPCR (RT-qPCR) was employed to examine transcript levels of some heat-responsive and defense genes. Total RNA extraction, first-strand cDNA synthesis, and qPCR assay were performed as described previously (Su et al., 2017). The tobacco *Actin2* was used as internal reference genes, and relative transcript levels were calculated as described by us (Zhang et al., 2019). Three biological replicates and three technical replicates were applied for the whole assays. The primes used for qPCR are listed in Supplementary Table S1.

### Statistical Analysis

The data were expressed as the means ± SE and subjected to statistical analysis using the SPSS (version 17.0, SPSS Inc., United States). Data were analyzed by one-way ANOVA, and means were compared by Duncan’s multiple range test at a significance level of *p* < 0.05. Histograms were plotted using the GraphPad Prism (v 8.01).

## Results

### Transcript Profiles of Two Tobacco *DOG1L* Genes in Response to ABA or Heat Stress

**Figure 1:**
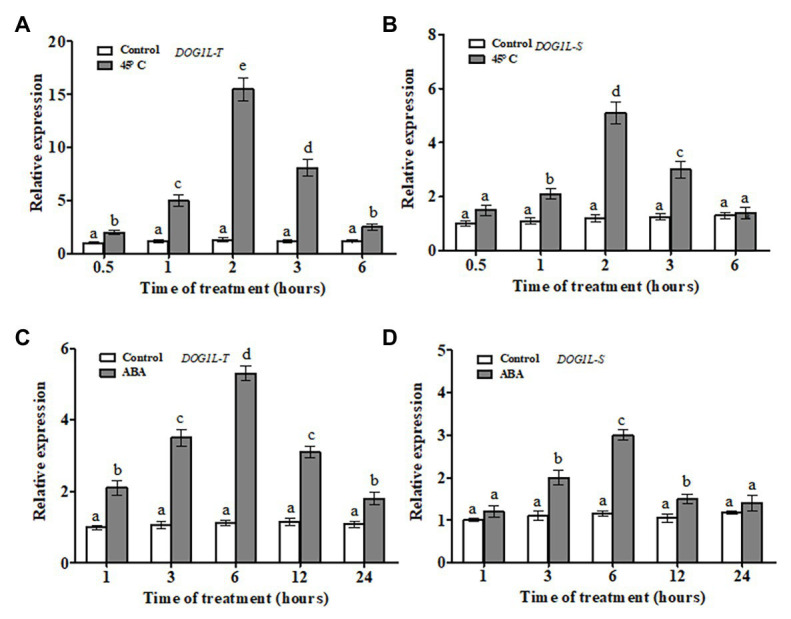
Transcript profiles of tobacco *delay of germination 1-like* (*DOG1L*) genes in response to abscisic acid (ABA) or heat stress. Two-week-old tobacco seedlings were treated with 45°C heat or 50 μM ABA, and seedlings were sampled at various time points after each treatment to extract RNA, and then transcript levels of both *DOG1L-T* and *DOG1L-S* were checked by quantitative PCR (qPCR) under heat stress **(A,B)** or ABA treatment **(C,D)**. For each reverse transcription quantitative PCR (RT-qPCR), the transcript levels of tobacco reference gene *Actin2* were also evaluated in various samples. For each experiment, three technical replicates were conducted. Data shown are Mean ± SE of three independent experiments. Statistical analysis was performed using ANOVA test (*p* < 0.05) and significant differences are indicated by different letters.

In our previous study, two homologs of the *DOG1L* genes (*NtDOG1L-T* and *NtDOG1L-S*) from the tetraploid tobacco *N. tabacum* (*Nt*, TTSS) were identified. Moreover, the two *DOG1L* genes shared high identity (98.7%) at amino acid level (Zhang et al., 2019). To further explore their responses to ABA or heat stress, time-course analyses of transcript levels of the two *DOG1L* genes in tobacco were conducted by RT-qPCR (Figure 1). Upon heat stress, the transcripts of both *DOG1L* genes increased significantly and peaked at 2 h until the end of the stress and then gradually decreased during 6 h of the heat stress (Figures 1A,B). Particularly, the *NtDOG1L-T* showed remarkable upregulation response (about 15-fold increase at 2 h of the stress). Similarly, both *DOG1L* genes responded alike during 24 h of the ABA treatment. Noticeably, the *NtDOG1L-T* expression increased rapidly after 1 h, and reached a peak at 6 h (about 5-fold increase), then gradually decreased, and ultimately maintained higher levels during the whole treatment (Figures 1C,D). These data imply that two *DOG1L* genes showed similar response pattern upon ABA or heat stress. Moreover, transcriptional response of the *NtDOG1L-T* was much stronger than that of the *NtDOG1L-S* during heat stress.

### Promoter Activity of the *NtDOG1L-T* in Response to ABA or Heat Stress

**Figure 2:**
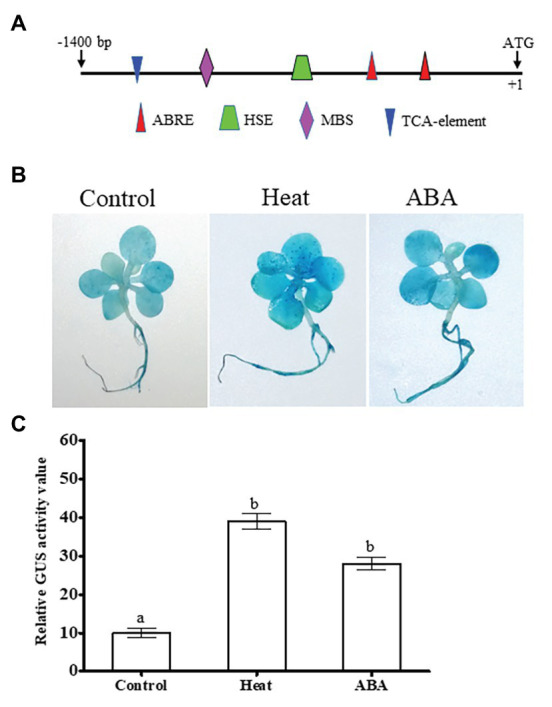
Promoter activity analysis of the tobacco *DOG1L-T* by histochemical staining under ABA or heat stress. **(A)** Promoter *cis*-regulatory elements of the tobacco *DOG1L-T*. Putative *cis*-elements in the promoter region of the *DOG1L-T* were predicted by PlantCARE. **(B)** β-glucuronidase (GUS) histochemical staining of tobacco seedlings transiently expressing the *DOG1L-T* promoter upon heat or ABA treatment. **(C)** Relative quantitative analysis of GUS activity by ImageJ. In **(B)** sterile cultured tobacco seedlings at 5- or 6-leaf stage were infiltrated by *Agrobacterium tumefaciens* harboring *DOG1L-T-Pro:GUS* construct. After 12 h the infiltrated seedlings were exposed to heat (45°C) or ABA (50 μM) treatment for 2 h, and then 6–8 seedlings for each treatment were collected for GUS histochemical staining. The seedlings grown in the solid MS medium were treated as controls. In **(C)** error bars represented SE of three replicates. Statistical analysis was performed using ANOVA test (*p* < 0.05) and significant differences are indicated by different letters.

To further understand its transcriptional responses to ABA or heat stress, we first isolated *NtDOG1L-T* promoter fragment (about 1.4 kb) and analyzed its regulatory elements using PlantCARE database.^2^ As shown in Figure 1A, in addition to a number of core *cis*-acting elements, such as TATA-box and CAAT-box elements, some known elements involved in hormone and abiotic stress responses were found in this region. Particularly, these elements contain two ABA response elements (ABRE,ACGTG; for ABA response), one heat shock response element (HSE,GAAXTTC; for heat response), one MYB binding site (MBS, CAACTG; for drought response), and one TCA-element (CAGAAAAGGA, for salicylic acid response; Figure 2A).

Histochemical detection of the *NtDOG1L-T* promoter activity was conducted with GUS staining in these heat- or ABA-treated seedlings. As shown in Figure 2B, transiently expressed *NtDOG1L-T-Pro:GUS* tobacco seedlings showed much stronger GUS staining upon heat or ABA treatment than control conditions (Figure 2B). These significant differences were also observed from the quantitative values in GUS staining intensity in these transgenic tobacco seedlings under heat, ABA, and control conditions (Figure 2C). These results indicate that transcription of the *NtDOG1L-T* is regulated by ABA or heat stress.

### Responses of Transgenic *NtDOG1L-T* Overexpression Lines to Heat Stress

**Figure 3:**
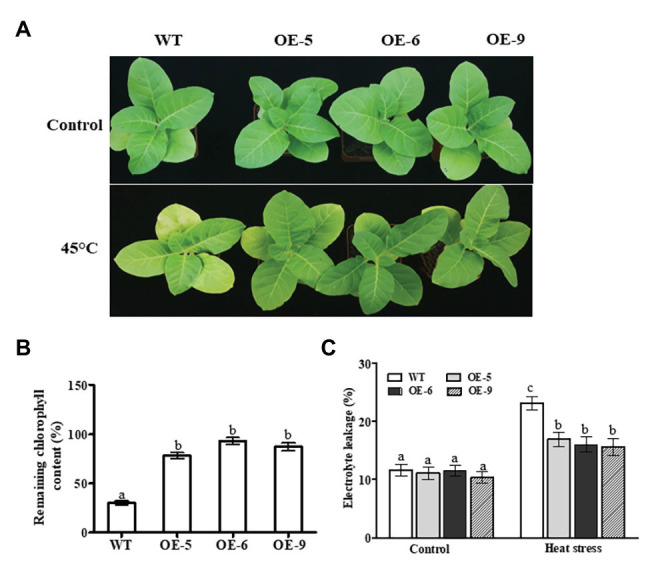
Phenotypic analyses of *DOG1L-T* transgenic tobacco lines under heat stress. **(A)** Phenotypes of 8-week-old wild type (WT) and transgenic lines subjected to a heat stress treatment (45°C) for 24 h. **(B)** Remaining chlorophyll contents in WT and transgenic lines after 24 h-heat stress. **(C)** Electrolyte leakage rates in transgenic lines and WT after 24 h-heat stress. The WT and transgenic lines (OE-5, OE-6, and OE-9) were cultured in MS plates under a 16/8 h light/dark cycle at 25°C in culture chamber for 2 weeks, and then the seedlings were transferred to pots filled with nutrient soil in growth room where they were regularly cultivated for additional 6 weeks. And then, 8-week-old WT and transgenic lines were transferred to a culture chamber and subjected to a heat stress treatment (45°C) for 24 h. The corresponding WT and transgenic lines grown in the other chamber at 25°C were treated as controls. In both **(B,C)** data are presented as means ± SE. Statistical analysis was performed using ANOVA test (*p* < 0.05) and significant differences are indicated by different letters.

We next examined the function of *NtDOG1L-T* in heat stress tolerance using the transgenic tobacco lines overexpressing *NtDOG1L-T* cDNA under 35S cauliflower mosaic virus (CaMV) constitutive promoter. Eight-week-old transgenic lines (OE-5, OE-6, and OE-9), along with WT tobacco plants were transferred to a culture chamber and subjected to a heat stress treatment (45°C). Meanwhile, the corresponding WT and transgenic lines grown in the other chamber at 25°C were treated as controls. After 24 h of treatment, the WT plants showed relative higher yellowing than these transgenic lines (Figure 3A). In contrast, under normal conditions, there were no obvious phenotypic differences between WT and transgenic lines (Figure 3A). Accordingly, remaining chlorophyll contents in these transgenic lines were significantly higher than that in WT plants (Approximately 56% on the average; Figure 3B). Furthermore, the electrolyte leakage rate in WT plants was about 45% higher than those in these transgenic lines (Figure 3C). These results evidenced that overexpression of *NtDOG1L-T* in transgenic overexpression lines improved tolerance to heat stress.

### Changes in Lipid Peroxidation and Antioxidant Enzyme Activities in *NtDOG1L-T* Transgenic Overexpression Lines Under Heat Stress

**Figure 4:**
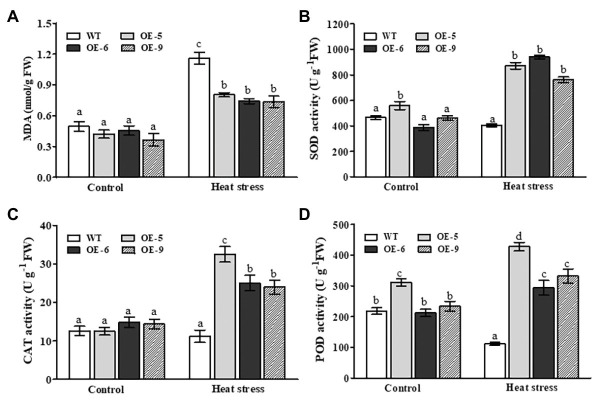
Changes in membrane peroxidation and antioxidant enzymes in the WT and *DOG1L-T* transgenic tobacco lines under heat stress. The malondialdehyde (MDA) content **(A)** and activity levels of antioxidant enzymes superoxide dismutase (SOD; **B**), catalase (CAT; **C**), and peroxidase (POD; **D**) in transgenic lines and WT plants upon 24 h-heat stress exposure. Statistical analysis was performed using ANOVA test (*p* < 0.05) and significant differences are indicated by different letters.

To uncover potential physiological mechanisms by which *NtDOG1L-T* improved heat stress tolerance, we measured MDA content and several antioxidant enzyme activities in *NtDOG1L-T* transgenic and WT plants under heat stress. MDA, the final product of membrane lipid peroxidation in plants, is an important parameter to reflect the potential antioxidant capacity of plant cells. Under control conditions, both transgenic and WT plants showed no significant differences in MDA content (Figure 4A). After heat stress, the MDA content in both transgenic and WT plants was significantly elevated, but the average increase magnitude of the MDA content in these transgenic lines was much lower than that in the WT (Figure 4A). This result indicated that these *NtDOG1L-T* transgenic overexpression lines showed less membrane damage than the WT upon heat stress.

We next assayed the activities of three antioxidant enzymes, SOD, CAT, and POD between *NtDOG1L-T* transgenic and WT plants upon heat stress. As shown in Figures 4B–D, after 24 h of heat stress, compared to their corresponding controls, activities of the three antioxidant enzymes in *NtDOG1L-T* transgenic lines markedly increased, but not in the WT plants. Moreover, these transgenic lines had more magnitudes of increases than the WT plants (Figures 4B–D). This result, along with fluctuations of MDA content, suggested that *NtDOG1L-T* overexpression activated several antioxidant enzymes and attenuated membrane damage upon heat stress in tobacco plants.

### Changes in Reactive Oxygen Species Levels in *NtDOG1L-T* Transgenic Overexpression Lines Under Heat Stress

**Figure 5:**
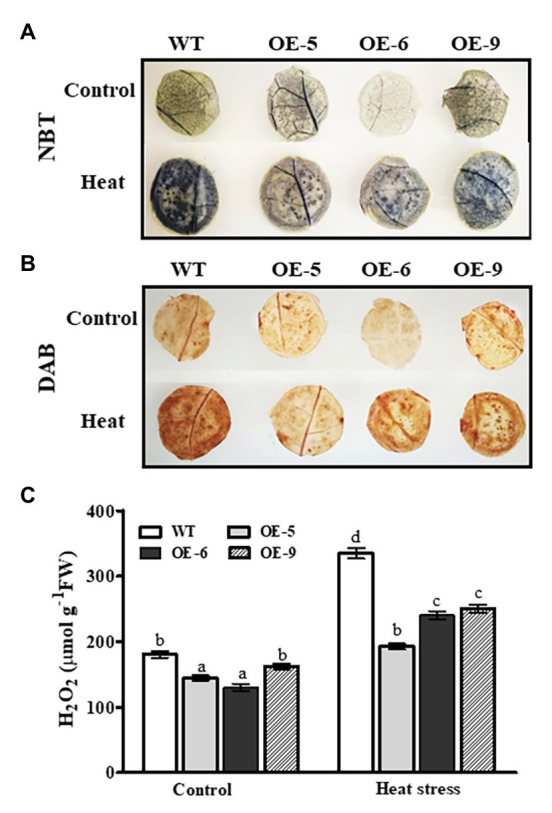
O_2_^−^ and H_2_O_2_ levels in the WT and *DOG1L-T* transgenic tobacco lines under heat stress. **(A)** O_2_^−^ production in leaf discs of WT and transgenic lines upon heat exposure. **(B)** H_2_O_2_ accumulation in leaf discs of WT and transgenic lines upon heat exposure. **(C)** Quantitative measurement of total H_2_O_2_ content in WT and transgenic lines upon heat exposure. The 8-week-old WT and transgenic overexpression lines were exposed to heat stress (45°C) for 24 h, and then, leaf discs from these stressed plants were taken for 3,3'-diaminobenzidine (DAB) and nitro blue tetrazolium (NBT) staining as described in the experimental procedures. Data are presented as means ± SE. Statistical analysis was performed using ANOVA test (*p* < 0.05) and significant differences are indicated by different letters.

To test the ROS scavenging activity of transgenic plants overexpressing *NtDOG1L-T*, accumulations of O_2_^−^ and H_2_O_2_ were examined upon heat stress. The 8-week-old WT and transgenic overexpression lines were exposed to heat stress (45°C) for 24 h, and then leaf discs from these stressed plants were sampled for DAB or NBT staining. As shown in Figures 5A,B, upon heat stress these three transgenic lines showed lower accumulations of O_2_^−^ and H_2_O_2_ than the WT in the leaf discs, as observed by NBT staining (brown pigment) and DAB staining (blue pigment; Figures5A,B). In contrast, under control conditions, there were no significant differences in O_2_^−^ or H_2_O_2_ accumulation between WT and transgenic lines, except for the line OE-6 (Figures5A,B). Furthermore, we quantified the differences in H_2_O_2_ content between transgenic lines and WT plants under heat or control conditions. As shown in Figure 5C, the three transgenic lines accumulated much lower H_2_O_2_ than the WT when exposed to 24 h of heat stress (Figure 5C). In addition, under control conditions, as shown in the Figure 5C, H_2_O_2_ content in the OE-6 was obviously lower than that in the WT (Figure 5C). These results, together with changes of antioxidant enzyme activities, indicated that *NtDOG1L-T* overexpression improved antioxidant enzyme activities, thereby reducing ROS accumulations and alleviating oxidative damage in transgenic overexpression lines under heat stress.

### Transcriptional Changes of Antioxidant- and Defense-Related Genes in *NtDOG1L-T* Transgenic Tobacco Lines Under Heat Stress

**Figure 6:**
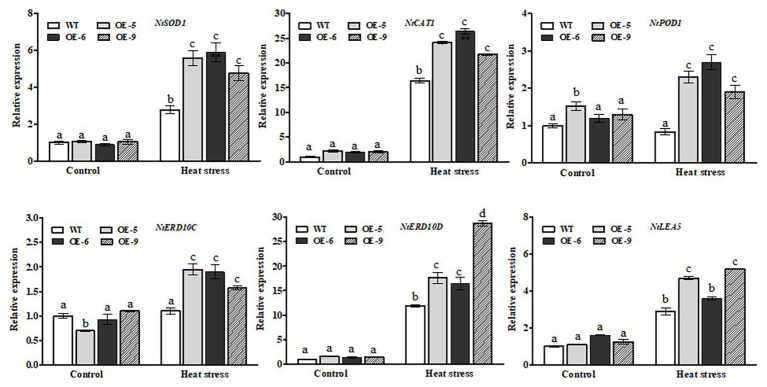
Transcriptional expression of antioxidant- and defense-related genes in WT and transgenic tobacco lines under heat stress. Eight-week-old WT and transgenic overexpression lines were exposed to heat stress (45°C) for 24 h, and leaves were sampled for RNA extraction, cDNA synthesis, and qPCR analysis. For each RT-qPCR, the transcript levels of tobacco reference gene *Actin2* were also evaluated in various samples. For each experiment, three technical replicates were conducted. Data shown are mean ± SE of three independent experiments. Statistical analysis was performed using ANOVA test (*p* < 0.05) and significant differences are indicated by different letters.

To elucidate possible molecular mechanisms of *NtDOG1L-T*-conferred heat tolerance, we used RT-qPCR assays to check transcript levels of several representative antioxidant- and defense-related genes between transgenic lines and WT plants under control or heat stress conditions. These genes included three antioxidant genes (superoxide dismutase-encoding gene *NtSOD1*, catalase-encoding gene *NtCAT1*, and cytoplasmic peroxidase *NtPOD1*) and three stress defense genes (early response protein 10-encoding genes *NtERD10C* and *NtERD10D*, and late embryogenesis abundant 5-encoding gene *NtLEA5*), which have been evidenced to play pivotal roles in protecting tobacco plants from abiotic stresses (Wang et al., 2019; Zhang et al., 2019). As shown in Figure 6-upper lanes, under heat stress transcript levels of the three antioxidant genes in the *NtDOG1L-T* transgenic lines were significantly upregulated, compared with those in the WT plants (Figure 6, upper lanes). In particular, the average expression level of the *NtPOD1* in these transgenic lines was about three times that of the WT plants (Figure 6, upper lanes). In contrast, under control conditions no clear differences were observed in these antioxidant genes, except for the *NtPOD1*, between WT and transgenic lines (Figure 6, upper lanes). Thereafter, we examined changes in expression levels of the three defense genes *NtERD10C*, *NtERD10D*, and *NtLEA5* between WT and *NtDOG1L-T* transgenic lines under heat stress. As shown in Figure 6-lower lanes, upon heat stress all the three defense-related genes in the transgenic lines showed significantly more transcripts than those in the WT plants (Figure 6, lower lanes). Noticeably, under control conditions these transgenic lines had significantly higher transcript levels of the *NtERD10D* than the WT plants (Figure 6, lower lanes). These data suggested that *NtDOG1L-T* overexpression upregulated the antioxidant- and defense-related gene expression upon heat stress.

### Transcriptional Changes of Heat- or ABA-Responsive Genes in *NtDOG1L-T* Transgenic Tobacco Lines Under Heat Stress

**Figure 7:**
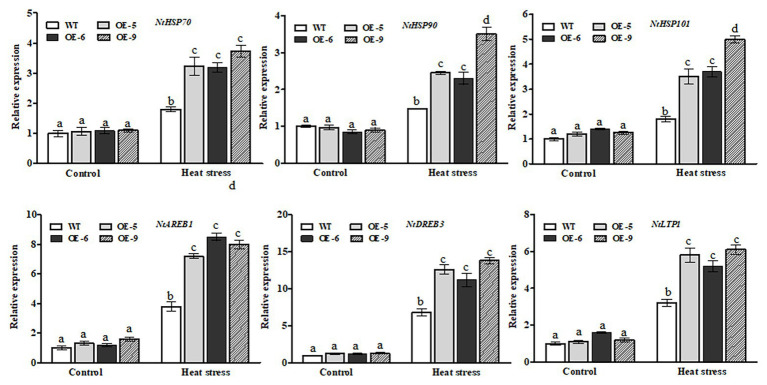
Transcriptional expression of heat- or ABA-responsive genes by qPCR in WT and transgenic tobacco lines under heat stress. Eight-week-old WT and transgenic overexpression lines were exposed to heat stress (45°C) for 24 h, and leaf samples were taken for RNA extraction, cDNA synthesis, and qPCR analysis. For each RT-qPCR, the transcript levels of tobacco reference gene *Actin2* were also evaluated in various samples. For each experiment, three technical replicates were conducted. Data shown are mean ± SE of three independent experiments. Statistical analysis was performed using ANOVA test (*p* < 0.05) and significant differences are indicated by different letters.

It is well known that HSPs, such as HSP70, HSP90, and HSP101, act as important molecular chaperones to protect many functional proteins from misfolding and aggregation during adaptation of plants to heat stress (von Koskull et al., 2007). Therefore, we performed RT-qPCR to examine the expression of these tobacco *HSP* genes between the *NtDOG1L-T* transgenic lines and WT plants upon heat stress. As shown in Figure 7-upper lanes, *NtDOG1L-T* transgenic lines exhibited significantly higher levels of transcripts for *NtHSP70*, *NtHSP90*, and *NtHSP101* than the WT upon 24 h of heat stress (Figure 7, upper lanes). In contrast, no significant differences were detected in expression levels of these three HSPs between WT and transgenic tobacco lines under control conditions (Figure 7, upper lanes).

Expression of the *NtDOG1L-T* was upregulated by exogenous ABA (Figures 1, 2), which promoted us to examine transcriptional changes in ABA-responsive genes between *NtDOG1L-T* transgenic lines and WT plants upon heat stress. These ABA-responsive marker genes included *AREB1* (encoding ABA response element-binding protein 1), *DREB3* (encoding dehydration-responsive element-binding protein 3), and *LTP1* (encoding lipid transfer protein 1), which have been shown to be involved in ABA or heat stress response (Xia et al., 2013, 2016; He et al., 2017). As shown in Figure 7-lower lanes, transcript levels of all the three ABA response genes in these *NtDOG1L-T* transgenic lines exhibited greater degrees of upregulation than the WT upon heat stress (Figure 7, lower lanes). These results indicate that *NtDOG1L-T* overexpression activated expression of the heat- and ABA-responsive genes under heat stress.

## Discussion

To date, most of the studies have been focused on roles of *Arabidopsis DOG1* family genes in important developmental processes, including seed dormancy and germination, flowering, and seed maturation (Bentsink et al., 2006; Dekkers et al., 2016; Huo et al., 2016). Recently, through transcriptome analyses, we have discovered a novel drought-responsive gene *NtDOG1L-T*, which encodes a DOG1-like protein and is involved in drought tolerance in tobacco (Zhang et al., 2019). In this study, we report molecular and physiological responses of the *NtDOG1L-T* transgenic overexpression plants under heat stress. Our results have demonstrated that *NtDOG1L-T* overexpression improves heat stress tolerance by regulating antioxidant metabolism and defense and heat stress response genes.

There are five *DOG1Ls* in *Arabidopsis* and six *DOG1Ls* in rice (Ashikawa et al., 2013). In tobacco, DOG1Ls are encoded by two genes (*NtDOG1L-S* and *NtDOG1L-T*) in the genome, which are highly homologous to the *Arabidopsis* DOG1-like 4 (DOGL4; Zhang et al., 2019). Previous studies have shown that DOG1 family proteins play important roles in various developmental processes, such as seed dormancy and germination, flowering, and seed development. In our study, we did not observe significant changes in seed dormancy and germination, and flowering time in *NtDOG1L-T* transgenic plants. Unexpectedly, we found that *NtDOG1L-T* overexpression promoted tobacco seedling growth (Zhang et al., 2019). In accordance with our observations, Sall et al. (2019) reported that reduced seed dormancy and longevity phenotypes were found in *dog1* seeds but not in *dogl4* mutants (Sall et al., 2019), suggesting that *DOG1L* genes might have functional divergence in seed dormancy function in *Arabidopsis*. In addition to developmental cues, *Arabidopsis DOG1* has been suggested to regulate drought tolerance (Yatusevich et al., 2017). Similarly, our recent research has found that overexpression of the *NtDOG1L-T* enhanced tolerance to drought stress in transgenic tobacco (Zhang et al., 2019). Furthermore, we uncovered the potential of *NtDOG1L-T* in heat stress tolerance in tobacco in this study. These data indicate that plant *DOG1Ls* have functional diversification.

Heat stress can induce excessive ROS accumulations, which are harmful and result in cell death. Thus, ROS homeostasis is tightly controlled in plants. CAT, POD, and SOD are three ROS-scavenging enzymes, which are necessary for ROS detoxification (Baxter et al., 2014). In our present study, *NtDOG1L-T* transgenic overexpression lines exhibited higher antioxidant enzyme activities and less H_2_O_2_ and O_2_^−^ accumulations than the WT during heat stress (Figures 4, 5). Furthermore, the transcripts of antioxidant-related genes *NtPOD1*, *NtSOD1*, and *NtCAT1* were significantly upregulated in the *NtDOG1L-T* transgenic plants during heat stress (Figure 6). These data suggested that *NtDOG1L-T* might enhance antioxidant capability for excess ROS scavenging by activating these antioxidant enzymes, thereby improving heat stress tolerance. Consistent with our conclusion, it was reported that overexpression of the *SOD* and *APX* genes in transgenic potato plants enhanced tolerance to heat and oxidative stresses (Kim et al., 2010). Similarly, Guan et al. (2013) evidenced that *Arabidopsis* copper/zinc superoxide dismutase 1 (SOD1) and SOD2 functioned as ROS scavengers to regulate thermotolerance (Guan et al., 2013). By contrast, impairment of APX or CAT decreased heat stress tolerance in *Arabidopsis* (Vanderauwera et al., 2011). Together, *NtDOG1L-T* conferred thermotolerance by enhancing antioxidant capability that efficiently maintained ROS homeostasis and reduced membrane damage under heat stress. In further study, it is needed to dissect the precise role of *NtDOG1L-T* in ROS homeostasis by multiple-omics approaches using these transgenic tobacco lines during heat stress.

Emerging evidence has shown that the phytohormone ABA plays a positive role in plant thermotolerance response (Larkindale et al., 2005; Huang et al., 2016; Suzuki et al., 2016). Our results indicate that *NtDOG1L-T* may be involved in ABA-mediated thermotolerance response in tobacco. This inference is based on the following three aspects: firstly, transcript levels of the *NtDOG1L-T* were rapidly induced by heat or ABA treatment, as revealed by qPCR (Figure 1). Secondly, there are two ABREs for ABA response and one HSE for heat stress response in promoter region of the *NtDOG1L-T* (Figure 2A). Furthermore, histochemical staining of the *NtDOG1L-T-Pro:GUS* transgenic tobacco seedlings showed that promoter activity of the *NtDOG1L-T* was markedly activated by ABA or heat stress (Figures 2B,C). These data suggest that transcription of the *NtDOG1L-T* gene is regulated by ABA or heat stress. Thirdly, transcript levels of ABA signaling genes *NtAREB1*, *NtDREB3*, and *NtLTP1* and heat-responsive genes *NtHSP70*, *NtHSP90*, and *NtHSP101* were significantly elevated in *NtDOG1L-T* transgenic lines upon heat stress (Figure 7), suggesting that the enhanced heat tolerance in the *NtDOG1L-T* transgenic plants might be associated with ABA signaling and HSP expression. In support of this notion, it was found that ABA conferred heat tolerance by inducing accumulations of several HSPs, including HSP70, HSP 90, and HSP101 in plants (Li et al., 2014). Interestingly, the *Arabidopsis DOG1* gene was also found to regulate seed development through genetically interacting with *ABI3* and modulating *ABI5* expression. Furthermore, transcriptome analysis revealed that hundreds of genes including *LEAs* and *HSPs* were altered in the *dog1* mutant (Dekkers et al., 2016). In addition, the transcription factors ABI3 and ABI5 have been evidenced to be involved in plant development, abiotic stress responses, and phytohormone crosstalk (Skubacz et al., 2016). Based on above reports plus our results, it is reasonable to speculate that *NtDOG1L-T* regulates thermotolerance response possibly by affecting ABA signaling, thereby activating downstream defense and heat stress-responsive gene expression in tobacco. Thus, it is of interest to explore exact role of the *NtDOG1L-T* in ABA signaling pathway during heat stress *via* proper tobacco mutants such as *abi3* and *abi5*.

**Figure 8:**
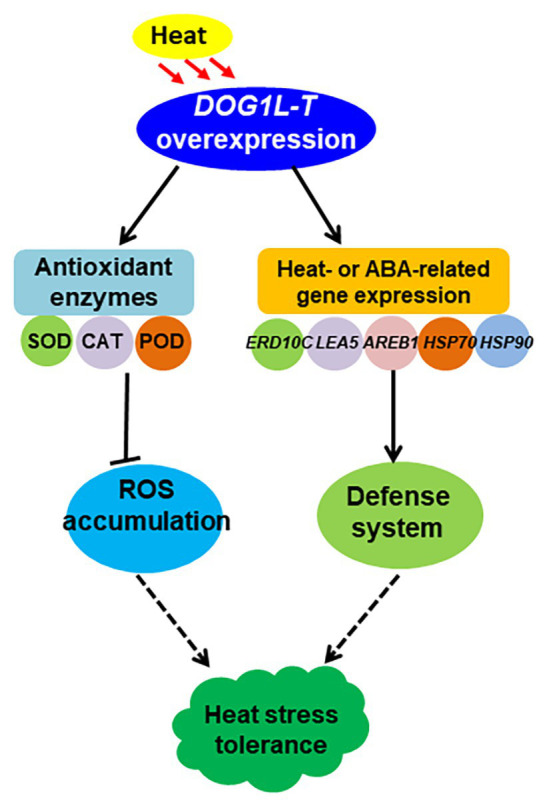
A model for the role of tobacco *DOG1L-T* under heat stress. *NtDOG1L-T* improves antioxidant machinery by activating the transcription of *SOD*, *CAT*, and *POD* when the plants are exposed to heat stress; meanwhile it upregulates expression of several heat- or ABA-responsive genes (*HSP70*, *HSP90*, *HSP101*, *LEA5*, and *ABRE1*). Thus, the *NtDOG1L-T* may play a protective role under heat stress in tobacco.

Together, our data have evidenced that *NtDOG1L-T* positively regulates heat stress tolerance at least partly due to increases of antioxidant capability and upregulation of the expression of defense-, heat-, and ABA-related genes in tobacco (Figure 8). This study provides valuable resource for the potential exploitation of *DOG1Ls* in genetic improvement of heat stress tolerance in crops.

## Data Availability Statement

The raw data supporting the conclusions of this article will be made available by the authors, without undue reservation.

## Author Contributions

ZX: Conceptualization, funding acquisition, supervision, and writing. YW, XD, SX, HL, and XZ: Investigation. XS, YC, TY, and ZX: Project administration. All authors contributed to the article and approved the submitted version.

### Conflict of Interest

XS, YC, and HL were employed by Sanmenxia Tobacco Company.

The remaining authors declare that the research was conducted in the absence of any commercial or financial relationships that could be construed as a potential conflict of interest.

## Footnotes

^1^http://rsb.info.nih.gov/ij/

^2^http://bioinformatics.psb.ugent.be/webtools/plantcare/html/
