# Supplementary material for: Overexpression of NtDOG1L-T Improves Heat Stress Tolerance by Modulation of Antioxidant Capability and Defense-, Heat-, and ABA-Related Gene Expression in Tobacco
Source: Front Plant Sci. 2020 Oct 30;11:568489. doi: 10.3389/fpls.2020.568489 (PMC7661468; doi:10.3389/fpls.2020.568489)
Supplement: Supplementary file 1 [file Data_Sheet_1.pdf]

Table S1. Primer sequences used in this study.

Reactions	Primer name	Sequence information	Enzyme site
Promoter expression	NtDOG1L-T-PF1	ACGAATTCTTCCTCATGCCAAAAGAGC	*Eco*RI
Vector construction	NtDOG1L-T-PR1	ACAAGCTTTGGTTTCTTTGGTCGGTC	*Hin*dIII
RT-qPCR	NtDOG1L-S-qF	GCAGGCGGTGAAGCTATTAG	
analysis	NtDOG1L-S-qR	CCACTGCGGATAAATTCCTAC	
	NtDOG1L-T-qF	GCAGGCGGTGAAGCTATTAG	
	NtDOG1L-T-qR	ACTCTATCGAGCCCAGTCAA	
	NtSOD1-qF	GACGGACCTTAGCAACAGG	
	NtSOD1-qR	CTGTAAGTAGTATGCATGTTC	
	NtCAT1-qF	TGGATCTCATACTGGTCTCA	
	NtCAT1-qR	TTCCATTGTTTCAGTCATTCA	
	NtPOD-qF	CTCCATTTCCATGACTGCTTTG	
	NtPOD-qR	GTTGGGTGGTGAGGTCTTT	
	NtERD10C-qF	ACGGACGAATACGGCAATC	
	NtERD10C-qR	TCTCCTTAATCTTCTCCTTCATCC	
	NtERD10D-qF	GAGGACACGGCTGTACCAGT	
	NtERD10D-qR	GCGCCACTTCCTCTGTCTT	
	NtLEA5-qF	TTGTTAGCAGGCGTGGGTAT	
	NtLEA5-qR	CTCTCGCTCTTGTTGGGTTC	
	NtHSP70-qF	CTTAGAAGGTTGAGAACTG	
	NtHSP70-qR	GGTAATGGTGGAGTAGAA	
	NtHSP90-qF	TGAGACTGCCCTCCTCACCT	
	NtHSP90-qR	ACCTCCTCCATCTTGCTACCC	
	NtHSP101-qF	GGCGATAGATTGCACCAAAGA	
	NtHSP101-qR	GCCCCAAGAAAAGGAATGAAC	
	NtAREB1-qF	GCAGCCATCTATCTATTC	
	NtAREB1-qR	GCAACTCATCCATATTCA	
	NtLTP1-qF	TTGCTGATTCGGCTTGGA	
	NtLTP1-qR	CTTCAACGGTCGCCTTCT	
	NtDREB3-qF	GCCGGAATACACAGGAGAAG	
	NtDREB3-qR	CCAATTTGGGAACACTGAGG	
	NtActin-qF	TGGCATCACACTTTCTACAA	
	NtActin-qR	CAACGGAATCTCTCAGCTCC	
	Nttubulin-qF	GCATCTTTGCGTACACTTTGCT	
	Nttubulin-qR	ACATAAGCCCAAAACTAGCTGGA	
